# Zoonotic Malaria: Non-*Laverania Plasmodium* Biology and Invasion Mechanisms

**DOI:** 10.3390/pathogens10070889

**Published:** 2021-07-13

**Authors:** Jing-Wen Hang, Farhana Tukijan, Erica-Qian-Hui Lee, Shifana Raja Abdeen, Yaw Aniweh, Benoit Malleret

**Affiliations:** 1 Immunology Translational Research Programme, Department of Microbiology and Immunology, Yong Loo Lin School of Medicine, Immunology Programme, Life Sciences Institute, National University of Singapore, Singapore 117545, Singapore; e0385049@u.nus.edu (J.W.H.); e0204997@u.nus.edu (F.T.); elqh18@nus.edu.sg (E.Q.H.L.); 2 Singapore Immunology Network (SIgN), Agency for Science, Technology and Research (A*STAR), Biopolis, Singapore 138648, Singapore; Shifana_Raja_Abdeen@immunol.a-star.edu.sg; 3 West Africa Centre for Cell Biology of Infectious Pathogens (WACCBIP), University of Ghana, Legon, Accra, Ghana; yaniweh@ug.edu.gh

**Keywords:** zoonotic malaria, host species, genome, pathology, invasion mechanism

## Abstract

Malaria, which is caused by *Plasmodium* parasites through *Anopheles* mosquito transmission, remains one of the most life-threatening diseases affecting hundreds of millions of people worldwide every year. *Plasmodium vivax*, which accounts for the majority of cases of recurring malaria caused by the *Plasmodium* (non-*Laverania*) subgenus, is an ancient and continuing zoonosis originating from monkey hosts probably outside Africa. The emergence of other zoonotic malarias (*P. knowlesi*, *P. cynomolgi*, and *P. simium*) further highlights the seriousness of the disease. The severity of this epidemic disease is dependent on many factors, including the parasite characteristics, host-parasite interactions, and the pathology of the infection. Successful infection depends on the ability of the parasite to invade the host; however, little is known about the parasite invasion biology and mechanisms. The lack of this information adds to the challenges to malaria control and elimination, hence enhancing the potential for continuation of this zoonosis. Here, we review the literature describing the characteristics, distribution, and genome details of the parasites, as well as host specificity, host-parasite interactions, and parasite pathology. This information will provide the basis of a greater understanding of the epidemiology and pathogenesis of malaria to support future development of strategies for the control and prevention of this zoonotic infection.

## 1. Introduction

Malaria is a life-threatening infectious disease, with approximately 200 million cases and 400,000 deaths annually worldwide [1]. This major public health burden is caused by infection with *Plasmodium* species transmitted by the female *Anopheles* mosquito.

**Figure 1:**
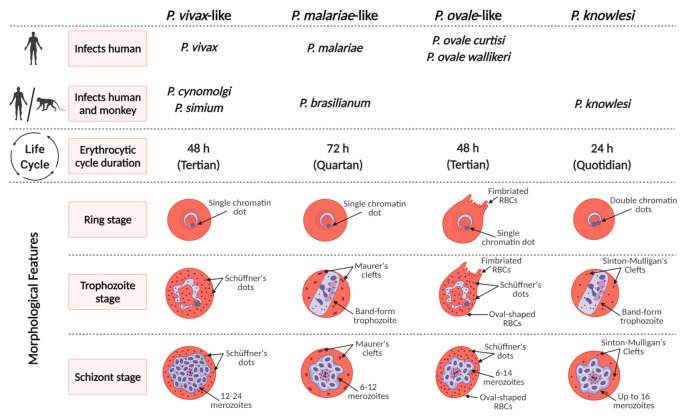
Characteristics of non-*Laverania Plasmodium* species in the erythrocytic cycle.

The life cycle of *Plasmodium* species involves three phases: the sexual sporogonic phase in the *Anopheles* mosquito vector, followed by the asexual pre-erythrocytic and asexual and sexual erythrocytic phases in a vertebrate intermediate host, such as humans and monkeys. Sporozoites are the infectious agents introduced into the intermediate hosts from the salivary glands of the female *Anopheles* mosquito during their blood meal. Once injected, the sporozoites migrate to the liver to infect hepatocytes and develop into liver schizonts (liver stage). When the hepatic schizont bursts, multiple merozoites are released and enter the bloodstream (blood stage) (Figure 1). During the erythrocytic stage, the merozoites invade erythrocytes via coupled receptor-ligand interactions. Several changes occur on the merozoite surface to facilitate the invasion process [2]. The infected erythrocyte is effectively hijacked to supply nutrients for parasite growth and to evade host immune responses [3]. After invasion, a parasitophorous vacuole is formed around the parasite. This process is followed by parasite development of ring stage trophozoites and replication to form mature schizonts. The number of merozoites released from the burst schizonts to invade new erythrocytes depends on the *Plasmodium* species. The clinical symptoms of malaria, especially fever, chills, and nausea, are linked with each round of merozoite release and erythrocyte destruction, and the blood cycle duration differs among *Plasmodium* species. During each blood stage replication, a small portion of the asexual parasites develop into sexual stage micro- or macro-gametocytes, with accelerated development under microenvironmental stress conditions such as high parasitemia, anemia, host immune responses, or drug treatment [4]. These gametocytes are responsible for the transmission of the disease following their uptake by the *Anopheles* mosquito during feeding, which will then undergo sexual reproduction and then multiple rounds of division to generate sporozoites (mosquito stage).

*Plasmodium* species are known to cause malaria in vertebrate hosts, which include humans, non-human primates, reptiles, birds, and rodents [5]. *Plasmodium* species are categorized into 14 subgenera based on their morphology and host range. *Plasmodium* species that infect humans and African apes are classified under the subgenus *Laverania* (*P. falciparum* is the species in humans). Of the non-*Laverania* species, four are specifically found in humans: *P. vivax*, *P. ovale wallikeri*, *P. ovale curtisi*, and *P. malariae*. Of the other species, *P. knowlesi*, *P. simium* [6], *P. brasilianum* [7], and recently *P. cynomolgi* [8] which have non-human primates as natural hosts, have also been found to infect humans naturally zoonotically. A list of non-*Laverania* primate-infecting *Plasmodium* species and their potential for zoonosis is provided in Table 1. The emergence of zoonotic malaria depends on several possible factors, including changes in human habitation patterns and ecology, the presence of susceptible *Anopheles* mosquitoes and human hosts in the same region, frequency of human-primate-mosquito contacts, vector adaptation (anthropophagic or zoophagic behavior), and potential genetic recombination between the human-infecting *Plasmodium* species and closely related non-human primate-infecting *Plasmodium* species resulting in increased virulence [9].

The emergence of zoonotic malaria represents a challenge to malaria control and elimination and further magnifies the seriousness of malaria as a global health concern. The severity of this epidemic disease is determined by the parasitic characteristics, host-parasite interactions, and pathology of each of these human-infecting species. Little is currently known about the biology and invasion mechanism of these parasites, which might enhance the potential of zoonosis continuation. In this review, we provide an overview of the literature describing the characteristics, distribution, and genomes of the parasites as well as their host specificity, host-parasite interactions, and pathology of infection by zoonotic malaria species in the non-*Laverania* clade. A better understanding of the epidemiology and pathogenesis of malaria will help to support the future development of strategies for the control and prevention of these zoonotic infections.

## 2. Geographical Distribution and Vertebrate Hosts of Zoonotic *Plasmodium* Species

The establishment of zoonotic malaria requires that the *Anopheles* vector has access to both monkey hosts and humans in the same area. The absence of any of these factors will impede the occurrence of zoonotic malaria. Recent progress in industrialization and extension of agricultural areas has led to extensive deforestation, leading to the loss of wildlife habitats and a displacement in animal populations, with monkeys’ groups living closer to human habitation. These conditions increase the risk of zoonotic malaria. Understanding the geographical distribution of the human-infecting *Plasmodium* species is important for the development of targeted strategies that increase the efficiency of measures for malaria control and elimination.

**Figure 2:**
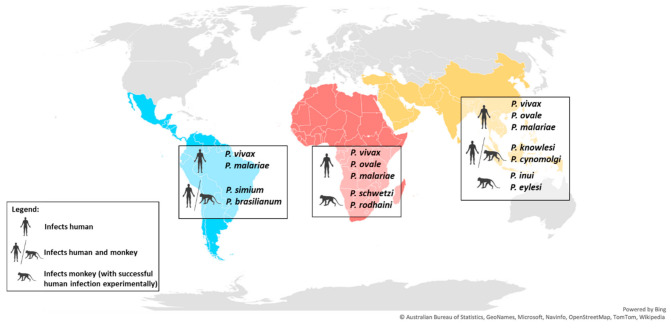
World map showing the distribution of the main non-*Laverania* human-infecting *Plasmodium* species.

Non-*Laverania Plasmodium* species are widely distributed throughout the world, and are particularly prevalent in tropical and sub-tropical regions of Southeast Asia. Infections also occur in sub-Saharan Africa, and Central and South America (Figure 2 and Table 1).

Among the non-*Laverania* subgenus, *P. vivax* is the most widespread human-infecting species and is prevalent in Southeast Asia, Central and South America, sub-Saharan Africa, and the Middle East. In 2018, 85% of the *P. vivax* infections were reported in only six countries: India (47%), Afghanistan (11%), Pakistan (8%), Ethiopia (8%), Papua New Guinea (6%), and Indonesia (5%) [1]. On the other hand, the cases of human infections by *P. ovale* and *P. malariae* are less frequent than the dominant *P. vivax*. *P. ovale* is predominantly distributed throughout sub-Saharan Africa, and occurs less frequently in Southeast Asia and the Middle East [10,11,12], while *P.* *malariae* is distributed widely across the tropical regions of sub-Saharan Africa, Central and South America, Southeast Asia, and the Middle East [6,10]. However, due to its characteristically low parasitemia [6], the prevalence of *P. malariae* infections is likely to be underreported.

In Southeast Asia, long-tailed and pig-tailed macaques are the most common hosts for the zoonotic *P. knowlesi* and *P. cynomolgi* [13,14] (Table 1). A study in Singapore, one of the two major cities with Rio de Janeiro with a tropical rainforest, has detected the presence of simian malaria in the wild macaques, which included mono- or co-infection of *P. coatneyi* (28.5%), *P. fieldi* (32.5%), *P. inui* (42.0%), *P. knowlesi* (47.5%), and the dominant *P. cynomolgi* (71.5%) [25]. On the other hand, the peridomestic macaques in Singapore are malaria-free, suggesting a low zoonotic transmission risk to the human population [25]. However, *P. knowlesi* and *P. cynomolgi* that are found only in Southeast Asia in their macaque natural hosts, were shown to infect humans zoonotically in the same region in 2004 and 2014, respectively [8,26,27,28,29,30,31]. The outbreak of *P. knowlesi* in Malaysia infers the high zoonotic malaria transmission risk in the areas where the natural macaque and human hosts and the *Anopheles* vectors co-exist. The similarities in the morphology of *P. malariae* and *P. knowlesi* in the erythrocytic stage resulted in misdiagnosis of this species in humans [29]. The morphology of *P. cynomolgi* is indistinguishable from that of *P. vivax* [13] and the species share 98% homology in the 18S RNA gene sequence [32]. These similarities increase the risk of misdiagnosis of *P. cynomolgi* as *P. vivax*. Asymptomatic human infection by *P. knowlesi* and *P. cynomolgi* have also been reported, raising difficulties in the evaluation of the distribution and prevalence of these parasites in humans [32]. Two other primate malarias that are found in Southeast Asia have been shown to be capable of infecting humans experimentally: *P. inui* naturally infects various species of the genus *Macaca* and *Presbytis* [13,14,17], while *P. eylesi* is found in *Hylobates lar* [13]. The ability of these parasites to infect humans may drive their zoonotic risk to the same region due to the sharing of natural hosts, and perhaps the *Anopheles* vectors with other human-infecting *Plasmodium* species.

Few cases of human infection by *P. vivax* have been reported in sub-Saharan Africa due to the Duffy-negative genotype that is predominantly found in African populations [33]. Studies have shown the importance of the interaction between the Duffy receptor for chemokine (DARC) and Duffy binding protein (DBP) in *P. vivax* and *P. knowlesi* invasion during the erythrocytic stage [34,35]. However, the ability of *P. vivax* to invade Duffy-negative erythrocytes was uncovered by the recent detection of *P. vivax* infection of Duffy-negative populations in African countries, such as Angola [36], Benin [37], Botswana [38], Cameroon [39], Ethiopia [40], Equatorial Guinea [36], Kenya [41], Madagascar [42], Mali [43], Mauritania [44], Senegal [45], Sudan [46], Uganda [47], and Namibia [48]. This might be due to a genotypic adaptation of this parasite that enhances its transmission. The receptor-ligand interactions involved in *P. vivax* and *P. knowlesi* invasion have not been fully elucidated; however, it is possible that the parasites utilize another route or receptor-ligand interaction, such as reticulocyte specific receptors [49,50], for successful invasion during the erythrocytic stage. The primate malaria *P. schwetzi* and *P. rodhaini* are found in sub-Saharan Africa where they infect African chimpanzees and gorillas [13,24]. The tertian malaria, *P. schwetzi*, has similar morphology to *P. vivax* and *P. ovale* [51,52], while *P. rodhaini* is closely related to *P. malariae* [13,51,52]. Both species can infect humans experimentally and because of their similarities with the human-infecting *Plasmodium* species, they have the potential for zoonotic transmission [13,24,53].

*P. simium* was reported to naturally infect New World monkeys including howler, woolly spider, and capuchin monkeys [6]. These monkeys are distributed in South and Central America, which is consistent with the reports of human *P. simium* infections that are restricted to the Atlantic Forest of the Southeast region of Brazil [6]. By contrast, *P. brasilianum* was shown to infect a large diversity of New World monkey species, covering the five families of these primates across regions of South America [20,54]. In terms of morphology, genetics, and immunological responses, the similarities between *P. simium* and *P. vivax*, and between *P. brasilianum* and *P. malariae*, have been confirmed [55,56,57,58]. The evolutionary and phylogenetic relationships between *P. vivax* and *P. simium*, and between *P. malariae* and *P. brasilianum*, have been evaluated on the basis of the genetic sequences of cytochrome b, merozoite surface protein-1, and SSU rRNA [59]. The similarities between these species have led to cases of misdiagnosis [6], which is further discussed in the next section.

## 3. Genome Characteristics of Zoonotic *Plasmodium* Species

Genomic research on *Plasmodium* species has undergone tremendous progress with the advances in technologies such as next-generation sequencing (NGS) that have overcome the challenges associated with obtaining sufficient gDNA of acceptable quality for whole genome shotgun sequencing (WGSS). The advent of NGS has facilitated the analysis of very small amounts of genetic material (5 *Plasmodium* genomes/μL) [60] without compromising the quality of the sequencing. This has led to a reduction in costs, making sequencing analyses more readily available. Consequently, the amount of information deposited in databases has increased over the years and several reference genomes of various *Plasmodium* species are now available. Public databases provide not only the sequences of human-infecting species of *Plasmodium*, but also genome information for zoonotic parasites such as *P. cynomolgi* and *P. knowlesi*. This knowledge has shed light on their evolutionary relationships. This, in turn, has provided important information that has clarified the genome diversity, host invasion strategies, and drug resistance mechanisms. Advances in single-cell sequencing techniques have facilitated the constant updating of reference genomes for each species with more accurate annotations. Comparative genomics, which provide an invaluable insight into the biology of the parasite and host-parasite interactions, will be covered in this review.

The genomes of the different *Plasmodium* species range from 20 to 35 megabases (Mb), with the nuclear genome distributed among 14 chromosomes (Table 2). Genomic comparisons of the different species showed variation in the arrangement of homologous genes on different chromosomes [61,62], which indicates a lack of synteny. However, it is important to note that *P. knowlesi*, *P. vivax,* and *P. cynomolgi* have highly conserved gene synteny along all 14 chromosomes despite several microsyntenic breaks [63]. This is expected as these species are from the same monkey malaria clade. While several comparative genomics have been conducted, these synteny maps tend to focus on human and rodent malaria. Hence, there is lack of synteny maps on zoonotic malaria which makes studying the zoonotic parasite traits and populations challenging. There are also marked variations in the guanine–cytosine (GC) content for the *Plasmodium* species. The GC-rich genes are also known to evolve faster than adenine–thymine (AT)-rich subtelomeric genes [64]. For the *P. knowlesi* chromosomes, the GC-rich repeat regions containing intrachromosomal telomeric sequences are found at multiple internal sites, arrayed tandemly or as components of larger repeat units [65]. This is unusual for *Plasmodium* species and these sequences appear infrequently *in P. vivax*, for example, while rodent malaria parasites have a relatively higher AT content of approximately 60%. Protein-coding exons have lower AT content than introns and intergenic noncoding regions. The rich AT content in the genomes of *Plasmodium* species often contributes to the technical hurdles in genomic sequencing [66]. A high AT content often reflects the existence of many low-complexity regions, microsatellites, and simple sequence repeats [67].

**Figure 3:**
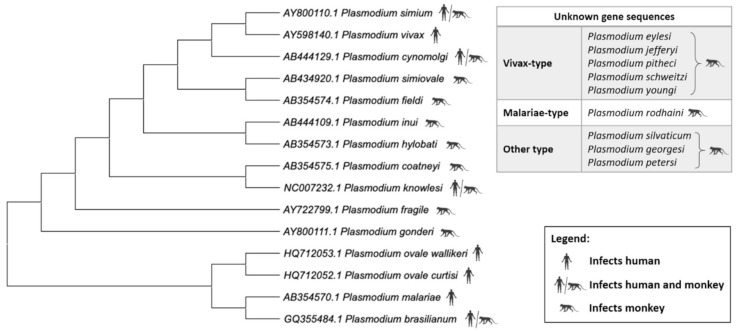
Phylogenetic tree of the *Plasmodium* species. Relationship of non-*Laverania Plasmodium* species based on mitochondrial cox3, cox1, and cytb sequences. Created using MegaX (Table S1).

*P. vivax* has a unique evolutionary path that is closely related to that of *P. cynomolgi*, *P. knowlesi*, and *P. simium* (Figure 3). A recent study which characterized 447 human *P. vivax* strains and 19 ape *P. vivax-like* strains has shown phylogenetic evidence that *P. vivax* is a sister clade of *P. vivax-like* instead of a lineage within the radiation of *P. vivax-like* [72]. Furthermore, the 19 *P. vivax-like* strains were categorized in two distinct clades which form a sister monophyletic lineage to the human *P. vivax*. These findings challenge previous studies which suggested that *P. vivax* is included inside *P. vivax-like* diversity [73,74]. Although *P. knowlesi* is closely related to *P. vivax* phylogenetically, there are distinct phenotypic differences such as the lack of hypnozoites (the latent hepatic form) in *P. knowlesi*. The first *P. knowlesi* to be sequenced was that of the H strain, Ok1 (A1) clone [65]. The nuclear genome of *P. knowlesi*, with a size of 24.4 Mb, contains genes that are approximately 80% orthologous to those of *P. vivax* [65]. A large proportion of the *P. vivax* genes were also found to be orthologous to *P. cynomolgi* genes. The orthologues identified had highly conserved genome positions [64] throughout all 14 chromosomes. Mitochondrial cytochrome oxidase I (mtCOI) gene amplicons were analyzed to detect *P. knowlesi* cases in Indonesian patients as it possesses a greater range of phylogenetic signal than any other mitochondrial and nuclear gene [75,76]. Phylogenetic analyses based on WGS of *P. simium* isolates indicated that *P. simium* is monophyletic within the broader diversity of *P. vivax* [68]. This suggested that *P. simium* first infected non-human primates as a result of a host-switch from humans carrying *P. vivax*. Large deletions within the coding region of reticulocyte-binding protein 2a (RBP2a) were observed in *P. simium* compared with *P. vivax* [68]. This is corroborated by another study conducted recently in which the authors observed host switching from humans to sylvatic monkeys in *P. simium*, indicating reverse zoonosis [77]. The study suggested the cause of the adaptation of *P. simium* to non-human primate hosts could be due to the >40% deletion of the coding sequence of PvRBP2a in the *P. simium* genome. Hence, the lack of PvRBP2a-mediated erythrocyte binding in *P. simium* may lead to a facilitation of alternative ligands for a more efficient monkey host cell infection [77]. While information about the genetic basis of *P. simium* zoonosis is scarce, the deletions suggest the existence of a possible facilitator of zoonotic transfer. As *P. simium* is almost indistinguishable from *P. vivax*, it is also possible that zoonotic transmission of *P. simium* has always existed, but the cases were misdiagnosed as *P. vivax*.

Due to low prevalence and parasite densities, *P. malariae* and *P. ovale* infections have not been widely discussed until recently. The improved sensitivity of the current molecular techniques has revealed an increasing number of mixed infections that include *P. malariae* and/or *P. ovale*. The nuclear genome of *P. malariae* was first sequenced from the CDC Uganda I strain [71]. The nuclear genome of *P. malariae* has a size of 33.6 Mb, with a GC content of approximately 24%. Interestingly, in this species almost all of its *Plasmodium interspersed repeats* (*pir*) genes, which may be important for antigenic variation, are pseudogenes. Phylogenetic analyses show that *P. ovale* is grouped together with *P. malariae*.

Approximately 40% of the total genes of *P. malariae* and *P. ovale* are subtelomeric [69]; however, the gene content of these subtelomeres differs greatly. The ancient divergence of the two *P. ovale* sub-species is corroborated by their discernible *pir* repertoires [69]. While the nuclear genomes of both *P. ovale wallikeri* isolates are approximately 35 Mb in size, a marked difference in the genome sizes of the *P. ovale curtisi* isolates was observed. The significant variation in the genome sizes of *P. ovale curtisi* 1 and *P. ovale curtisi* 2 was found to be due to differences in the magnitude of the *pir* gene family repertoires represented within the genome [71]. Both *P. ovale* species have a significantly larger genome size than the zoonotic *Plasmodium* species which are highlighted in this review (Table 1). This could be due to the expansion of variant gene families. In both *P. ovale* species, the *surfin* gene family contains more genes. The roles of *surfin* gene products are predicted to be erythrocyte surface receptors as they are exported to host erythrocyte cytoplasm and then exposed on the erythrocyte surface [78]. The SURFIN genes in *P. malariae* and both *P. ovale* species contain schizont-infected cell agglutination (SICA) domain. Since SICAvar proteins are known to have an evolutionary link with SURFIN proteins in *P.* *vivax* [79], the differential expansion of *surfin* genes in both *P. ovale* species (125 genes in *P. ovale wallikeri* and 50 genes in *P. ovale curtisi*) could be useful for discerning the species at the molecular level.

The simian parasite *P. brasilianum* was first reported in New World monkeys. Investigations in the 1960s demonstrated that humans could be experimentally infected with *P. brasilianum* from monkeys and vice versa [13]. However, it was only recently reported that naturally acquired infections of *P. brasilianum* occur in humans [7]. *P. brasilianum* is morphologically indistinguishable from *P. malariae* and their genomes are very similar [69,80], with 99% similarity when aligned [80]. Hence, this could pose a problem in the use of molecular diagnostic techniques to distinguish between these two species with confidence and it is possible the recent unexpected detection of *P. malariae* in Costa Rica could be due to a misdiagnosis of *P. brasilianum*. The close genetic relationship of *P. brasilianum* and *P. malariae* subtypes suggests that both species constitute a complex group that has diverged as a result of allopatric speciation.

## 4. Characteristics and Pathology Associated with Individual Zoonotic *Plasmodium* Species

Among the non-*Laverania* subgenus that infect humans, *P. vivax* and *P. ovale* are similar morphologically and phenotypically. Like *P. vivax*, *P. ovale* infects reticulocytes preferentially whereas *P. malariae* invades senescent erythrocytes [81,82,83]. Many studies have shown that *P. malariae* exhibit normocyte cell tropism, although in vitro culture is still problematic [84]. *P. malariae*-infected cells are usually smaller than uninfected erythrocytes. It is possible that *P. malariae* causes acceleration of the maturation of the infected erythrocytes, leading to the mature erythrocyte phenotype of infected cells as demonstrated for reticulocytes with *P. vivax* [85]. This phenomenon may lead to the erroneous conclusion that *P. malariae* infects senescent normocytes preferentially.

The typical characteristic of *P. ovale* is the stippling or Schüffnerization [13], which appears in the early blood stages of *P. ovale* and intensifies as the parasite grows. Schüffnerization in *P. ovale* is also more marked than that in *P. vivax* [13]. Interestingly, caveola–vesicle complexes, which are similar to those of *P. vivax*, are also present in *P. ovale*-infected erythrocytes. These complexes consist of caveolae surrounded by vesicles in an alveolar fashion and form along the host cell plasmalemma. The fact that these complexes have not been reported in *P. malariae* suggests that a caveola–vesicle complex corresponds to a Schüffner’s dot. *P. malariae* induces the production of spikes, caveolae, electron-dense material, vesicles, clefts, and knobs in the erythrocyte [86,87]. The vesicles and caveolae, which may be involved in antigen trafficking, do not form the caveola–vesicle type complexes that are seen in *P. vivax*-infected erythrocytes [86].

Deformation of erythrocytes is required to allow their passage through the microvasculature and the sinusoids of the spleen. *P. vivax* increases the deformability of the infected erythrocytes, which facilitates their passage through the narrow inter-endothelial slits of the splenic sinusoids. Nonetheless, *P. vivax*-infected erythrocytes do sequester [88] and avoid splenic clearance [89]. Recently, it has been observed that intact *P. vivax*-infected erythrocytes accumulate in the spleen. Splenic tropism is the highest in P. vivax infections with more than 98% of asexual-stage parasites. This may suggest that chronic malaria could be due to the infection of the spleen, instead of blood infection solely [90,91]. Infected erythrocytes deformability can also be observed in *P. ovale* and *P. cynomolgi* infections [87].

As mentioned previously, it is highly implied that *P. brasilianum* and *P. simium* are a zoonotic transfer from *P. malariae* and *P. vivax*, respectively. It is therefore no surprise that some of the morphologies of the zoonotic *Plasmodium* species are similar to those of *P. vivax*, *P. malariae*, and *P. ovale*. The characteristics and pathology of *P. knowlesi*, *P. cynomolgi*, *P. simium*, and *P. brasilianum* are discussed below.

### 4.1. Plasmodium knowlesi

Naturally acquired human *P. knowlesi* infections were thought to be extremely rare until a large focus of human infections was reported in Malaysia Borneo in 2004 [29]. Before the advent of molecular detection methods, *P. knowlesi* infections were often misdiagnosed as *P. malariae* due to morphological similarities [92]. Double-chromatin dots in early trophozoites and no enlargement of infected erythrocytes are the morphological features of *P. knowlesi* [92]. The band-form trophozoites of *P. knowlesi* greatly resemble those of *P. malariae*; however, minor differences can be observed between *P. knowlesi* and *P. malariae*, mainly, in the double-chromatin dots in trophozoites and the number of merozoites produced. These minor differences are often overlooked during microscopic diagnosis especially when only thick blood films are examined because parasitemia are low. The median peak parasitemia observed in knowlesi malaria is ca. 2500/μL in humans, but can reach much higher levels in severe cases [93]. *P. knowlesi*-infected erythrocytes also show ‘Sinton and Mulligan’ stipplings [13]. Some of the ultrastructural features of *P. knowlesi*-infected erythrocytes include lack of knobs and the presence of caveolar pits without vesicles association in the infected erythrocytes during the trophozoite stage [94,95]. This is unlike *P. vivax* infection, in which caveola–vesicle complexes and clefts are formed in the erythrocytes [95]. These structures are involved in protein trafficking from the vivax parasite to the erythrocyte membrane [86,87]. Cleft-like structures and tubular vesicles features that wrap around regions of the host cell cytoplasm are also observed in knowlesi malaria. Electron tomography analysis revealed budding of small vesicles from the Sinton Mulligan’s clefts [94]. Generally, the prepatent period ranges from 9 to 12 days in humans and from 7 to 14 days in *Macaca fascicularis* [13,96].

*P. knowlesi* has the shortest asexual replication cycle of all *Plasmodium* species leading to rapidly increased parasite levels. Most human *P. knowlesi* cases are chronic and symptomatic with a potentially fatal outcome [97,98,99]. Thrombocytopenia is the most reported condition in the majority of these cases [92,98,100,101]. Furthermore, knowlesi malaria patients may be hyperparasitemic and develop hepatorenal/renal dysfunction, with jaundice, hypoglycemia, and lactic acidosis being the potentially devastating metabolic consequences [102]. Other clinical findings include respiratory distress with increased permeability of the pulmonary capillaries [102]. Similarly, vivax malaria is known to cause acute respiratory distress syndrome (ARDS) in which pulmonary vascular sequestration and post-treatment alveolar-capillary inflammation are observed [103].

The pathogenesis of severe *P. knowlesi* infection is not yet fully understood. In one study, a *P. knowlesi*-infected patient died within 2 h of admission to hospital and was found to have multiorgan failure [98]. An accumulation of infected erythrocytes was found in this patient, indicating possible sequestration of parasites and hemorrhagic complications in vital organs, though chronic inflammatory infiltrates were not present. The kidney showed signs of acute tubular necrosis and prominent endothelial cell infiltration was observed in heart sections [98]. Complicated knowlesi malaria cases often had higher levels of cytokines such as tumor necrosis factor alpha (TNF-α), IL-6, IL-8, IL-1ra, and IL-10 [104]. This is similar to vivax malaria in which high plasma levels TNF-α and IFN-γ are correlated with disease severity. However, plasma concentrations of IL-10 were shown to be inversely related to disease severity of vivax malaria [105]. While further investigations are needed to fully understand the pathology of severe *P. knowlesi* infection, studies have shown that the pathogenesis of complicated disease is different from that of *P. vivax*.

### 4.2. Plasmodium cynomolgi

*P. cynomolgi* was first discovered in 1907 in long-tailed macaques (*Macaca fascicularis*) imported into Germany from Java [13]. The first case of a naturally acquired human infection with *P. cynomolgi* was reported in the Malay Peninsular in 2014 [8]. More recently, asymptomatic human infections with both *P. knowlesi* and *P. cynomolgi* were discovered in western Cambodia [32]. As a zoonotic species, *P. cynomolgi* has a high degree of morphological and biological similarities with *P. vivax*, making it hard to distinguish the two species [8,32]. Therefore, it can be speculated that some *P. cynomolgi* infections have been misdiagnosed as *P. vivax*. Due to these similarities, *P. cynomolgi* has been used as a model for *P. vivax* [106]. In the early stages, enlargement of the infected erythrocytes becomes noticeable as the young parasite grows to a diameter of approximately half more than that of the original host cell. Eventually, Schüffner’s stippling and pigments become more prominent as the parasite progresses through the stages of development [13]. The late trophozoite stage of *P. cynomolgi* closely resembles that of *P. vivax* [13] with the presence of Schüffner’s dots in both trophozoites and schizonts as for *P. vivax* [107,108]. At maturity, the number of merozoites ranges from 14 to 20, with an average of 16 [13]. The duration of the erythrocytic stage cycle of *P. cynomolgi* is 48 h, with a prepatent period of 19 days observed in humans. In *M. speciosa* and *M. mulatta*, the prepatent period ranges from 7 to 16 days [13]. The two most studied strains of *P. cynomolgi* in its experimental host *Macaca mulatta* are the Mulligan or M strain and the subspecies *P. c. bastianellii* or the B strain. The mean parasitemia for the B strain is higher than that of the M strain [13].

During primary infections in rhesus macaques, mild to severe anemia is observed, with mild thrombocytopenia observed in all infected macaques [109]. Accidental laboratory-acquired human infections with *P. cynomolgi* resulted in clinical symptoms including anemia, leucopenia, thrombocytopenia, elevated erythrocyte sedimentation rate, hypoalbuminemia, and hyperglobulinemia [110]. The patients with the naturally acquired human infection with *P. cynomolgi* experienced non-specific symptoms that mimicked a flu-like syndrome [8]. In a recent case of a naturally acquired human infection, the patient experienced muscle pain, fever, headache, and abdominal pain with low-grade parasitemia. The different parasitic stages observed in the patient’s blood smears resembled *P. vivax* with slight morphological differences [111]. It has long been established that *P. cynomolgi* is a relapsing malaria in macaques [13,112]. However, to date, no studies have been reported on any relapse cases in naturally acquired human infections.

### 4.3. Plasmodium simium

*P. simium* was first identified in 1951 in a monkey from the state of São Paolo in Brazil [113]. It is similar at the morphological, genetic, and immunological levels to *P. vivax* [6]. However, the trophozoites of *P. simium* are reported to be less amoeboid, with coarser, and more prominent Schüffner’s dots than *P. vivax* [6]. The Schüffner’s stippling is very distinct in all stages, except, in the young ring stage and can be seen filling up the entire host cell [13]. Young erythrocytes infected with late developmental forms of *P. simium* often display multiple surface clefts and cytoplasm-associated microvesicles (Schüffner’s dots) [13,58]. Dual infections are quite common in simium malaria. The mature schizonts of *P. simium* produce 12 to 18 merozoites. The asexual blood stage cycle is approximately 48 h [13]. In squirrel monkeys, the mean parasitemia can be greater than 10,000 per µL by day 8 and may remain at this level or higher for 25 days.

Patients with naturally acquired *P. simium* infection reported clinical symptoms that are consistent with those of *P. vivax* malaria. These patients responded successfully to chloroquine and primaquine, with no relapses or deaths [6]. The pyrogenic threshold of *P. simium* infection is extremely low [6], although the reason for this is still unknown. Further studies are required to establish whether *P. simium* is capable of producing hypnozoites and in turn, causing relapse.

### 4.4. Plasmodium brasilianum

*P. brasilianum* was first described in monkeys in the early 20th century. To date, no consistent morphological, immunological, or genetic differences between *P. brasilianum* and *P. malariae* have been identified [7,56,114]. *P. brasilianum* causes quartan malaria as it has a 72 h erythrocytic cycle [13]. In malaria-endemic regions, co-infections of *P. brasilianum* with other *Plasmodium* species are common. In areas of South America where both humans and monkeys co-exist, differentiating *P. malariae* and *P. brasilianum* infections is impossible due to their high degree of morphological similarity [115]. Since parasitemia in the human host is low and parasite counts rarely exceed 50 per µL [13], co-infections are often under-detected by microscopy. Ziemann’s stippling can be observed during the late ring stage [13]. Once mature, the schizonts release between 8 and 12 merozoites. Similarly, *P. malariae* has a production of relatively low number of merozoites (6 to 14, with an average of 8) per erythrocytic cycle [13,115]. *P. brasilianum* infection induces the formation of knobs, short and long clefts, and electron-dense material—all of which can also be observed in *P. malariae*-infected erythrocytes. Spikes, which are superficially similar to knobs, are also present in great quantity on RBCs infected with *P. brasilianum* and *P. malariae*. While these spikes are also present in *P. ovale*-infected RBCs, they are usually lesser and bigger in size [116]. These structures appear to contain different *P. brasilianum* antigens, indicating that each structure functions independently in trafficking *P. brasilianum* protein to the erythrocyte surface [86].

During the course of an infection in *Ateles* monkeys, the parasitemia may rise and fall following a predetermined pattern. An initial rise in parasite numbers is followed by a significant reduction, a low-grade blood infection and finally, short periods of sub-patent parasitemia interspersed with spontaneous recrudescence [13]. Recrudescence can occur when the host is subjected to stressful conditions or becomes immunocompromised [117]. While the infection in *Ateles* and *Saimiri* monkeys can continue for at least 249 days, the parasitemia in humans infected with *P. brasilianum* did not exceed 27 days. On the other hand, there is a wide range of prepatent period in naturally transmitted *P. malariae,* ranging from 18 to 59 days in humans [13]. The clinical manifestations were generally milder than observed in infections with *P. cynomolgi* or *P. knowlesi* [13]. *P. brasilianum* can induce renal pathology when it persists as a chronic infection in humans [117].

## 5. Role of *Plasmodium* Ligands in Erythrocytic Invasion

Successful infection of vertebrate hosts by *Plasmodium* species depends on several factors, one of the main determinants being the presence of appropriate erythrocyte surface receptors. The two types of infected erythrocytes are reticulocytes and normocytes. There is variation in the levels of surface receptors expressed by erythrocytes at different stages of differentiation, which lead to the differences in the cell tropism for *Plasmodium* parasites invasion. *P. vivax* [118], *P. ovale wallikeri* [82], *P. ovale curtisi* [82], *P. cynomolgi* [119], and *P. simium* [120] have strict reticulocyte-restriction in humans. By contrast, *P. malariae* [83] and *P. brasilianum* [120] invades only senescent normocytes, while *P. knowlesi* [121] invades normocytes with a preference for reticulocytes.

To date, *P. knowlesi* is the only zoonotic form of malaria for which continuous in vitro culture in human erythrocytes has been established [122]. In the human blood circulation, reticulocytes account for only 1 to 2% of the adult peripheral blood and mature to form normocytes over a 72 h period. This poses a challenge to maintaining an in vitro cultivation of parasites that invade only reticulocytes. Conversely, *P. malariae* that invades normocytes has difficulties in establishing its long-term culture with adult peripheral blood, which has made its cell tropism a controversy. The difficulties in establishing continuous parasite culture hinder the identification of the receptor-ligand interactions involved in the merozoite invasion mechanism.

**Figure 4:**
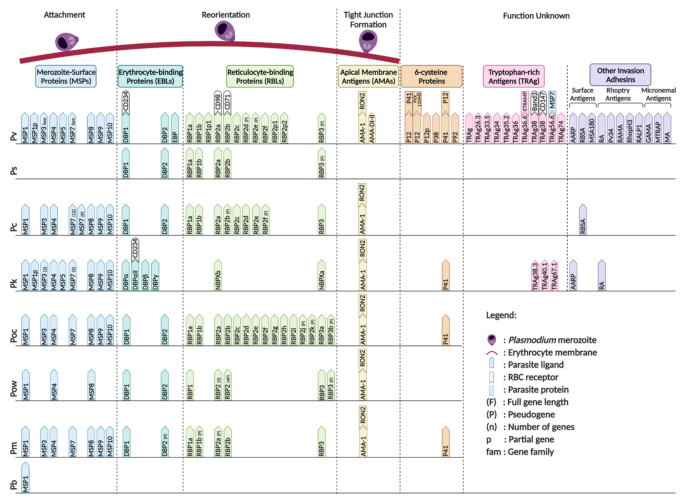
Non-*Laverania Plasmodium* species ligands with their respective erythrocyte receptors during invasion in erythrocytic stage. (F) = full gene length; (P) = pseudogene; (n) = number of genes; p = partial genes; fam = gene family. Created with BioRender.com.

The basic structure of merozoites is similar across the different *Plasmodium* species; hence, all may utilize the same invasion mechanism during their erythrocytic stage despite having different cell tropism patterns and receptor-ligand interaction preferences [123] (Figure 4). As merozoites are released from schizonts, invasion of erythrocytes through a series of events is required to initiate a new cycle of erythrocytic development. This process is initiated by reversible attachment of the merozoite to an erythrocyte, which is thought to be mediated by glycosylphosphatidyl inositol (GPI)-anchored merozoite surface proteins (MSPs), although the specific receptor-ligand interaction has not yet been fully elucidated [123]. The merozoite then undergoes apical reorientation, which has been shown to trigger different interactions involving the reticulocyte-binding-like (RBPs) and erythrocyte-binding-like (EBPs) proteins. These interactions initiate the formation of a tight and irreversible attachment between the merozoite and the erythrocyte. The formation of the tight junction has been shown to involve members of the rhoptry neck proteins (RON) family, specifically RON2, and apical membrane antigen 1 (AMA1) [124]. After tight junction formation, the merozoites penetrate the erythrocyte via glideosome activity and finally seal themselves within the parasitophorous vacuole.

The recognition and initial attachment of merozoites to the erythrocyte surface is governed by the MSPs on the merozoite surface. Several MSPs have been identified, with MSP1, MSP3, MSP4, MSP5, MSP6, MSP7, MSP8, MSP9, and MSP10 being commonly found in all the non-*Laverania* human-infecting *Plasmodium* species, except *P. simium*, which is less well characterized. PvMSP1 has been proved to have highly specific binding affinity towards reticulocytes, and has similar cleavage sites to those of PfMSP1 [125]. Cleavage at these sites generates 42- and 19-kDa carboxy-terminal fragments that are believed to be involved in initial attachment to the erythrocyte and the invasion process [125]. *P.* *vivax* merozoite surface protein 1 paralog (PvMSP1P) has been reported and hypothesized to play an important role in parasite adhesion based on its ability to bind erythrocytes via its 19-kDa C-terminal region [126]. MSP3 gene family members confer the antigenic properties on the parasite and contribute to the immune response induced in non-human primates against erythrocytic stage *Plasmodium* parasites [64]. It has been demonstrated that MSP7 in *P. falciparum* binds to MSP1 on the merozoite surface and the invasion efficiency has been shown to be reduced by MSP1 knockout [64,127]. The significant expansion of the MSP3 and MSP7 gene families in *P. vivax* compared with *P. falciparum* might account for its improved immune evasion and adhesion properties [64]. The other MSP gene families do not have any known function in the invasion mechanism.

RBPs and EBPs are the two major ligand families that account for the cell tropism of the parasites. A total of 11 PvRBP family members have been identified through homology analysis of *P. vivax* RBP and *P. yoelli* Py235 member gene sequences [128]. The 11 Pv-RBPs contain five full-length genes, three partial genes and three pseudogenes [128]. PvRBP2a has been shown to bind CD98 on reticulocytes with high affinity [129]. Cryo-electron microscopy (cryo-EM), immunoprecipitation, and fluorescence resonance energy transfer (FRET)-based assays further confirmed the specific binding of PvRBP2b to CD71 [130,131]. Gruszczyk et al. showed that the PvRBP2b binding ability is directly proportional to the level of CD71 expression on the erythrocyte surface [131]. The interaction of PvRBP2a and PvRBP2b with CD98 and CD71, respectively, on reticulocytes is the basis of the strict reticulocyte cell tropism of *P. vivax*. The RBP-encoding genes of *P. ovale curtisi* and *P. ovale wallikeri* have expanded to 15 and 12 copies, respectively, with several identified as pseudogenes [71,132]. *P.* *cynomolgi* is known to infect reticulocytes and also has seven full-length RBPs (PcRBP1a, PcRBP1b, PcRBP2a, PcRBP2c, PcRBP2d, PcRBP2e, and PcRBP3) and two fragmented RBPs (PcRBP2b and PcRBP2f) [132]. On the other hand, five PsRBPs, comprising four full-length RBPs and one pseudogene have been reported in *P. simium* [68]. The *Plasmodium* species that invade normocytes have been shown to possess fewer copies of RBPs compared with the reticulocyte-invading *Plasmodium* species, which highlights the importance of the RBPs in determining *Plasmodium* cell tropism. The senescent normocyte-invading *P. malariae* appears to have three full-length RBPs (PmRBP1a, PmRBP2b, and PmRBP-3) and two pseudo RBPs (PmRBP1b and PmRBP2a) [132]. *P.* *knowlesi*, which invades both normocytes and reticulocytes, possesses only two full-length RBPs, PkNBPXa [133] and PkNBPXb [132]. The limited number of RBPs in these normocyte-invading parasites indicates the presence of alternative receptor-ligand interactions that facilitate the invasion process.

EBPs are released to the merozoites surface from the microneme during apical reorientation of the merozoite [127]. The EBPs that have been discovered in the non-*Laverania Plasmodium* species are members of the Duffy binding protein (DBP) family, with DBP1 and DBP2 being the most commonly identified forms [68,69,132,134,135]. However, *P. knowlesi* has three DBP copies (DBPα, DBPβ, and DBPγ) [132]. CD234, which is also known as the Duffy antigen receptor for chemokines (DARC), is utilized by *P. vivax* and *P. knowlesi* for their invasion into erythrocytes via interaction with DBP1 and DBPα, respectively [136]. Following CD234 knockout, the invasion ability of *P. vivax* was reportedly reduced [137]. However, it has recently been reported that the Duffy-null population in Africa is infected with *P. vivax* [36,37,38,39,40,41,42,43,44,45,46,47]. DBP1 has been shown to expand from three to eight copies in Duffy-null-infected cells [134], leading to the hypothesis that the expansion of DBP1 facilitates binding to other erythrocyte receptors with low affinity to achieve successful invasion [134]. Moreover, the failure of Pv-DBP1 to bind to Duffy-null erythrocytes, and the binding of PvDBP2 to Duffy-positive and Duffy-null erythrocytes at low frequency suggests that PvDBP2 may have a role in the invasion mechanism when PvDBP1 is non-functional [134].

During tight junction formation, AMA1 is secreted to the merozoite surface while RON complexes are injected to the erythrocyte surface via the rhoptry neck. AMA1 is known to bind to RON2 and both are found in all the species discussed in this review, with the exception of *P. simium* and *P. brasilianum*, for which there is no evidence to date. *P. vivax* AMA1 domains I and II (Pv-AMA1_DI-II) are responsible for the specific targeting of CD71^+^ reticulocytes [138].

The 6-cysteine protein family comprises a group of conserved surface proteins expressed throughout the life cycle of the *Plasmodium* parasites in *Anopheles* mosquito and vertebrate hosts. Five of these proteins have been discovered in *P. vivax*: PvP12 [139], PvP12p [140], PvP38 [141], PvP41 [142], and PvP92 [140]. PvP12 is co-localized with PvRON2 in the rhoptry of *P. vivax* merozoites [139]. The immunogenicity of PvP12 discovered in a *P. vivax*-infected patient indicates that it plays a role in *P. vivax* invasion [139]. PvP41 is usually released from the merozoite surface in the schizont stage of the erythrocytic cycle [142]. PvP38 is found on the schizont during the erythrocytic cycle [141]. PvP12 and PvP41 form a complex on the merozoite surface and interact with PVX_110945, which is an uncharacterized protein [140]. PvP92 has been suggested to be responsible for preventing complement-dependent lysis of merozoites as its *P. falciparum* orthologue (PfP92) is known for this function [143]. P41 has also been identified in *P. knowlesi*, *P. ovale curtisi*, and *P. malariae* [144].

Tryptophan-rich antigens (TRAgs) have been recommended as malaria vaccine subunit candidates based on the ability of some members to bind to erythrocytes [145,146,147]. TRAgs have been identified in two *Plasmodium* species, *P. vivax,* and *P. knowlesi*. In *P. vivax*, 10 TRAgs were found to have the ability to bind to human erythrocytes (PvTRAg, PvTRAg26.3, PvTRAg33.5, PvTRAg34, PvTRAg35.2, PvTRAg36, PvTRAg36.6, PvTRAg38, PvTRAg69.4, and PvTRAg74) [147]. PvTRAg36.6 and early transcribed membrane protein (ETRAMP) were found to be co-localized in the apical region of the *P. vivax* merozoite in the early erythrocytic stage, indicating a functional role for their interaction in the development or maintenance of the parasitophorous vacuole membrane [148]. The interaction of PvTRAg38 with host erythrocytes is known to facilitate parasite growth. PvTRAg38 interacts with basigin and band 3 via the P2 and P4 regions (amino acid positions 167–178 and 197–208), respectively [149,150]. On the other hand, PvTRAg56.2 has been found to interact with PvMSP7 and co-localize with PvMSP1 on the merozoite surface, indicating that it might be involved in surface protein stabilization [148]. In contrast to *P. vivax*, *P. knowlesi* has been found to possess PkTRAg38.3, PkTRAg40.1, and PkTRAg67.1 that bind to human erythrocytes [151].

Other malarial proteins that remain poorly characterized have been proposed as potential malaria vaccine candidates. In *P. vivax*, these include the surface antigens AARP [152], RBSA [153], and MSA180 [154]; the rhoptry antigens P34 [140], RAMA [155], RhopH3 [156], RALP1 [157], and RA [158]; and the micronemal antigens GAMA [159,160], MTRAP [140], and MA [140]. Furthermore, the surface antigens RBSA [153] and AARP [152] were also identified in *P. cynomolgi* and *P. knowlesi*, respectively, and the rhoptry antigen RA [158] was identified in *P.* *knowlesi*. Most of these antigens are found on *P. vivax*, the most prevalent non-*Laverania Plasmodium* species. This suggests their possible role in the host-parasite interactions, pathology, and immunology in better adaptation to the human host. Plasmodium antigens can be identified based on their orthologs in other human infected *Plasmodium* species, such as *P.* *falciparum* [64]. By targeting these conserved regions, it is possible to generate a vaccine candidate for pan-malaria therapies, or species-specific therapies due to the multiple and diverse antigens across different families in all the *Plasmodium* species.

## 6. Conclusions

Eliminating malaria remains a challenge that is heightened with the rise in zoonotic infections. In recent years, more non-*Laverania Plasmodium* species, which were previously thought to infect only non-human primates, have been shown to be also capable to infect humans naturally (*P. knowlesi*, *P. cynomolgi*, *P. simium*, and *P. brasilianum*) and experimentally (*P. eylesi*, *P. inui*, *P. schwetzi*, and *P. rodhaini*). The emergence of host-switching relies on the presence of human hosts, monkey natural hosts, and the *Anopheles* vectors in the same region. The *Anopheles* vectors play a role in transmitting the parasites to either host, resulting in a change in the pattern of parasite infection. However, this review focuses only the *Plasmodium* species in human hosts. While the characteristics of these zoonotic species are similar, the development of molecular tools have greatly reduced the risk of zoonotic misdiagnosis, especially in developing countries where malaria is endemic. Thus, anti-malarial drug discovery should also concern all *Plasmodium* species and not only be focused on *P. falciparum*, in order to maximize the likelihood of achieving malaria elimination/eradication [161]. A comprehensive understanding of the genetic variability, molecular evolution, invasion mechanisms, and pathogenesis of these zoonotic species may provide insights that can be used to reduce the zoonotic risk and develop strategies for the control and elimination of existing zoonoses.

## Figures and Tables

**Table 1:** Geographical distribution, human infectivity, and natural host(s) of non-*Laverania Plasmodium* species. ● = Species infecting humans naturally; ● = species infecting humans experimentally; ● = species that fail to infect humans; ● = unknown human infectivity.

Parasite Distribution	*Plasmodium* Species	Human Infection	Natural Host(s)	References
**Asia** **Africa** **America**	*P. vivax*	●	Human (*Homo sapiens*)	[1]
	*P. malariae*	●	Human (*Homo sapiens*)	[6,10]
**Asia** **Africa**	*P. ovale*	●	Human (*Homo sapiens*)	[10,11,12]
**Asia**	*P. coatneyi*	●	Long-tailed macaque (*Macaca fascicularis*)	[13,14]
	*P. cynomolgi*	● ●	Long-tailed macaque (*Macaca fascicularis*), Pig-tailed macaque (*Macaca nemestrina*), Bonnet macaque (*Macaca radiata*), Formosanrock macaque (*Macaca cyclopis*), Rhesus macaque (*Macaca mulatta*), Silvered leaf monkey (*Presbytis cristatus*), Hanuman langur (*Presbytis entellus*)	[13,14]
	*P. eylesi*	●	Lar gibbon (*Hylobates lar*)	[13]
	*P. fieldi*	●	Long-tailed macaque (*Macaca fascicularis*), Pig-tailed macaque (*Macaca nemestrina*), Bonnet macaque (*Macaca radiata*), Rhesus macaque (*Macaca mulatta*), Baboon (*Papio doguera*)	[13,14]
	*P. fragile*	●	Bonnet macaque (*Macaca radiata*), Toque macaque (*Macaca sinica*)	[13,15,16]
	*P. hylobati*	●	Silvery gibbon (*Hylobates moloch*)	[13]
	*P. inui*	●	Cynopithecus niger (*Macaca nigra*), Formosanrock macaque (*Macaca cyclopis*), Long-tailed macaque (*Macaca fascicularis*), Rhesus macaque (*Macaca mulatta*), Pig-tailed macaque (*Macaca nemestrina*), Bonnet macaque (*Macaca radiata*), Silvered leaf monkey (*Presbytis cristatus*), Dusky leaf monkey (*Presbytis obscurus*)	[13,14,17]
	*P. jefferyi*	●	Lar gibbon (*Hylobates lar*)	[13]
	*P. knowlesi*	●	Long-tailed macaque (*Macaca fascicularis*), Pig-tailed macaque (*Macaca nemestrina*), Black-crested Sumatran langur (*Presbytis melalophos*)	[13,14]
	*P. pitheci*	●	Orangutans (*Pongo pygmaeus*)	[13,18]
	*P. simiovale*	●	Toque macaque (*Macaca sinica*)	[13]
	*P. silvaticum*	●	Orangutans (*Pongo pygmaeus*)	[18]
	*P. youngi*	●	Lar gibbon (*Hylobates lar*)	[13,19]
**America**	*P. brasilianum*	● ●	Capuchin monkey (*Cebus albifrons*, *C. apella*, *C. tapucinus*, *C. c. tapucinus*, *C. c. imitator*), Spider monkey (*Ateles fusciceps*, *A. geoffroyi*, *A. g. geoffroyi*, *A. g. grisescens*, *A. panistus*, *A. p. paniscus*, *A. p. chamek*), Squirrel monkey (*Saimiri sciurea*, *S. boliviensis*), Woolly monkey (*Lagothrix cana*, *L. infumata*, *L. lagotricha*), Howler monkey (*Alouatta fusca*, *A. palliata*, *A. seniculus straminea*, *A. villosa*), Bald uakari (*Cacajao calvus*), Woolly spider monkey (*Brachyteles arachnoides*), Titi monkey (*Callicebus moloch ornatus*, *C. torquatus*)	[13,14,20]
	*P. simium*	● ●	Black howler monkey (*Alouatta fusca*), Woolly spider monkey (*Brachyteles arachnoides*), Capuchin monkey (*Cebus and Sapajus* spp)	[6,13,14,21]
**Africa**	*P. georgesi*	●	Mangabey (*Cercocebus albigena*, *C. galeritus agilis*)	[22]
	*P. gonderi*	●	Mangabey (*Cercocebus galeritus agilis*, *C. aterrimus*, *C. atys*), Drill (*Mandrillus leucophacus*)	[13,14,22]
	*P. petersi*	●	Mangabey (*Cercocebus albigena*)	[23]
	*P. rodhaini*	●	Chimpanzee (*Pan troglodytes*)	[13]
	*P. schwetzi*	●	Chimpanzee (*Pan troglodytes*), Gorilla (*Gorilla* spp.)	[13,24]

**Table 2:** Comparison of genome features of zoonotic *Plasmodium* species.

	Species	*Pv* P01	*Pv* Sal1	*Pc* B	*Ps* Howler	*Pk* H	*Pow*_1	*Pow*_2	*Poc*, Nigeria 1	*Poc*_2	*Pm* UG01	*Pb* Bolivian 1
Features												
**Total length (Mp)**		29	26.8	26.2	29	24.4	35.2	35.1	34.5	38	33.6	30
**No. of scaffolds**		374	374	1663	2192	28	1378	1593	4015	2227	7270	953
**GC%**		39.7	42.3	40.4	44.6	37.5	28.91	29.12	28.46	27.76	24.74	24.8
**Total no. of chromosomes**		14	14	14	14	14	14	14	14	14	14	14
**No. of protein coding genes**		6642	5433	5776	-	5188	8582	8813	7950	8825	6540	6050
**References**		[68,69]	[69]	[70]	[68]	[65,69]	[71]	[71]	[71]	[71]	[69,71]	Accession number: GCA_001885115.2

*Pv* = *P. vivax*; *Pc* = *P. cynomolgi*; *Ps* = *P. simium*; *Pk* = *P. knowlesi*; *Pow* = *P. ovale wallikeri*; *Poc* = *P. ovale curtisi*; *Pm* = *P. malariae*; *Pb* = *P. brasilianum*.

## Data Availability

All data are available in the review.

## Supplementary Materials

The following are available online at https://www.mdpi.com/article/10.3390/pathogens10070889/s1, Table S1: title. Non-*Laverania Plasmodium* species mitochondrial gene sequences.
